# The intention to get COVID-19 vaccine and vaccine uptake among cancer patients: An extension of the theory of planned behaviour (TPB)

**DOI:** 10.1007/s00520-022-07238-5

**Published:** 2022-06-25

**Authors:** Rocco Servidio, Antonio Malvaso, Deborah Vizza, Moira Valente, Maria Rosita Campagna, Melania Lo Iacono, Leslie R. Martin, Francesco Bruno

**Affiliations:** 1 Department of Cultures, Education and Society, University of Calabria, Arcavacata di Rende Cosenza, Italy; 2 Neurology Unit, IRCCS San Raffaele Scientific Institute, Milan, Italy; 3 Academy of Cognitive Behavioral Sciences of Calabria (ASCoC), Lamezia Terme, Italy; 4 Voluntary Association “Ali Rosa”, Rende, CS Italy; 5 Department of Psychology, La Sierra University, 4500 Riverwalk Parkway, Riverside, CA 92515 USA; 6 Regional Neurogenetic Centre (CRN), Department of Primary Care, ASP Catanzaro, Lamezia Terme, CZ Italy; 7 Association for Neurogenetic Research (ARN), Lamezia Terme, CZ Italy

**Keywords:** Cancer patients, Theory of planned behaviour, COVID-19, Vaccine hesitancy, Vaccine uptake

## Abstract

The psychosocial impact of coronavirus disease 2019 (COVID-19) on human life is well-known. Although vaccine protection represents an effective way to control the spread of the virus, vaccination hesitancy may decrease individuals’ willingness to get vaccinated, including among cancer patients. Therefore, the objective of the current study was to examine the predictors of cancer patients’ intentions to receive COVID-19 vaccinations and vaccine uptake, using and integrating the theory of planned behaviour (TPB) and the health belief model (HBM). A sample of 276 Italian cancer patients (54% female and 46% male) ranging from 19 to 85 years (*M* = 49.64, *SD* = 11.53) was recruited by administering an online questionnaire. The current study results showed that cancer patients with higher trust in health authorities tended to have vaccine-positive subjective norms, perceived that vaccination was under their control, and viewed COVID-19 vaccines positively. On the other hand, the perceived risk of COVID-19 was related to subjective norms but not to perceived behavioural control or attitudes towards COVID-19 vaccination. The current study reveals that TPB variables can function effectively as mediators between perceived risk, trust, and intention to vaccinate but at different levels. Together, these findings suggest that effective interventions (both public health messaging and personal medical communications) should focus on enhancing trust in health authorities, while at the same time endeavouring to highlight subjective norms that are vaccine-positive.

## Introduction

The coronavirus disease 2019 (COVID-19) pandemic has impacted the physical [1] and personal mental health [2], as well as the individual and social well-being [3], and educational systems [4], among others. However, it has posed even greater risks for vulnerable groups, including cancer patients [5, 6]. Cancer patients have a higher risk of contracting COVID-19 [7] and experience more severe COVID-19 symptoms than the general population [8, 9], likely due to the immunosuppressive effects of cancer and its medical treatments [10], as well as the comorbidities experienced by this group [11]. Furthermore, findings show evidence of increased risk of COVID-19 mortality among cancer patients [12, 13], highlighting the urgent need to protect them from the transmission of COVID-19.

The threat posed by COVID-19, and the need for mass vaccination, came at a time of growing vaccine scepticism — reluctance or refusal to vaccinate despite the availability of the vaccine — in developed countries (World Health Organization, 2020). Even before the COVID-19 pandemic, the World Health Organization (2020) ranked hesitation about vaccines as one of the top ten global health threats.

Private and public health systems worked quickly to develop vaccines to protect the world population against COVID-19 [14], but this scientific effort raised concerns about safety for some individuals. These concerns were then accentuated by anti-vaccination messages in mass media and social media [15]. Furthermore, data demonstrated high rates of hesitancy about the COVID-19 vaccine [16, 17], which jeopardised immunisation efforts [18, 19], and these problems have not fully abated. In addition, preliminary studies identified this high rate of COVID-19 vaccine hesitancy even in cancer patients [20–23] and survivors [24]. Therefore, it is crucial to understand which factors can predict individuals’ intention to accept COVID-19 vaccinations, particularly for more vulnerable groups such as cancer patients.

The theory of planned behaviour (TPB) represents a consolidated conceptual framework [25] that has been recently applied to COVID-19 vaccine hesitancy and uptake [26]. The TBP states that behavioural intentions are the most proximal determinants of behaviour and that three factors converge to predict behavioural intentions [27]: attitudes, subjective norms, and perceived behavioural control. The first factor, attitudes, represents the individual’s attitudes towards behaviour and reflects whether opinions about the behaviour are favourable or unfavourable. The second factor, subjective norms, describes the perceived expectations of significant others and motivation to conform to these expectations. The third factor is perceived behavioural control or the perception of how easy or difficult it is to perform the behaviour. According to the TBP, these three factors combine in an interactive (not additive) way to determine behavioural intention which, in turn, governs individual behaviour (although perceived behavioural control can also directly influence behaviour). The results of a recent meta-analysis indicated that the TPB model explained vaccine hesitancy and behavioural factors related to vaccination intention [28], and other studies have demonstrated that TPB predicts intentions to get the COVID-19 vaccine [26, 29]. A more complete understanding of COVID-19 vaccine-related behaviour would add to these model additional variables that might further enhance the predictive efficacy of the TPB.

The aim of the present study was to evaluate the factors affecting the intention to get a COVID-19 vaccine among cancer patients, by integrating the TPB and the health belief model (HBM). HBM [30] is one of the most widely used models for examining the relationship between health behaviour and the use of health services and particularly in the context of influenza vaccination [31, 32]. Specifically, one of the main constructs of HBM is perceived risk (i.e. the individual’s perceived susceptibility to, and the level of threat associated with, a particular threat), which has been considered as an essential psychological determinant in predicting health behaviour [e.g. [33]. Indeed, individuals who perceive a particular risk, such as COVID-19 infection, are assumed to adopt more preventive health behaviour to avoid or minimise health risks [34]. Therefore, understanding these psychological determinants of vaccine hesitancy for cancer patients and the possible factors influencing risk perception is essential for formulating communication strategies and policies to promote vaccination among cancer patients. Another promotive determinants, trust in health authorities, has been highlighted as a factor that may decrease individuals’ perceptions of vaccination risks [35, 36], and a lack of trust in health authorities is a salient factor in shaping COVID-19 vaccination hesitancy [37]. Indeed, results from a recent scoping review showed that, among others, perceived risk and trust in health authorities were associated with higher COVID-19 vaccine acceptance in the general population [38]. Moreover, past vaccination habits have also been found to predict intentions to get vaccinations and the COVID-19 vaccine in particular [39].

**Fig. 1:**
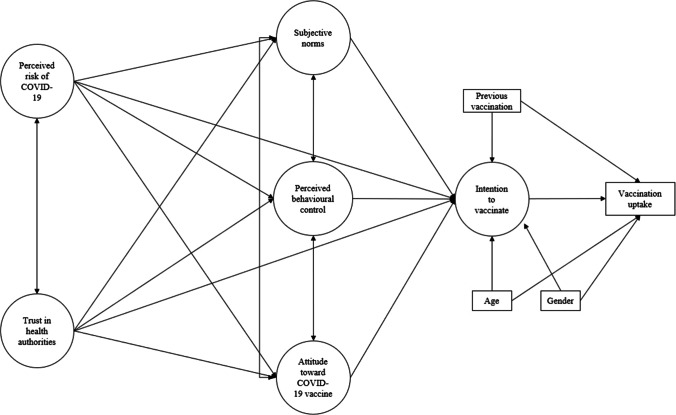
The proposed theoretical model predicting intention to vaccinate and vaccination uptake in a sample of cancer patients

However, to the best of our knowledge, few studies have combined the TPB and HBM models to explore health-related behaviours and intention to receive influenza vaccine among the general population [e.g. [38]. Additionally, there is little to no discussion of a relationship between TPB and HBM, among cancer patients. Therefore, in line with the scientific evidence discussed above, we designed a research model (Fig. 1) to examine predictors of cancer patients’ intentions to receive COVID-19 vaccinations and vaccine uptake. Specifically, we hypothesised that (1) perceived risks of COVID-19 and trust in health authorities should positively predict the TPB determinants of the behaviour (attitudes, subjective norms, and perceived behavioural control) and, in turn, the intention to receive the COVID-19 vaccine; (2) the relationship between perceived risk of COVID-19, trust in health authorities, and the intention to receive a COVID-19 vaccine should be mediated by TPB variables (subjective norms, perceived behavioural control, and attitudes towards COVID-19 vaccine); and (3) the intention to get a COVID-19 vaccination should positively predict COVID-19 vaccine uptake.

## Method

### Participants

 The sample was composed of 276 cancer patients (54% female and 46% male) ranging in age from 19 to 85 years with a mean age of 49.64 years (*SD* = 11.53). The socio-demographic and clinical characteristics of participants are shown in Table 1. Slightly more than 50% of the sample (145 participants) took part in the follow-up.

**Table 1:** Sample socio-demographic and clinical characteristics

	*N*	%
Sex		
Female	149	54%
Male	127	46%
Age group, years		
< 40	48	17.3%
40–60	180	65%
61–70	42	15.2%
71–85	7	2.5%
Educational levels		
Primary school	5	1.8%
Secondary school	33	11.2%
High school	136	49%
University	103	37.2%
Marital status		
Single	31	11.2%
In a relationship	43	15.5%
Married	159	57.4%
Divorced	36	13%
Widowed	8	2.9%
Occupation (after diagnosis)		
Homemaker	31	11.2%
Employed outside the home	155	56%
Unemployed	36	13%
Student	9	3.2%
Retired	46	16.6%
Tumour		
Breast	70	25.3%
Uterus/ovaries	49	17.7%
Prostate	39	14.1%
Skin	27	9.8%
Lung	24	8.7%
Stomach/intestines	19	6.9%
Lymphatic system	16	5.8%
Kidney	11	4%
Multisite	5	1.8%
Brain	4	1.4%
Thyroid	4	1.4%
Pancreas	3	1.1%
Blood	3	1.1%
Bladder	2	0.72%
Liver	1	0.3%
Tumour stage, UICC		
I	82	29.6%
II	94	34%
III	72	26%
IV	29	10.4%
Surgery		
Yes	234	84.5%
No	43	15.5%
Radiation therapy		
Yes	118	42.6%
No	159	57.4%
Chemotherapy		
Yes	172	62.1%
No	105	37.9%
Hormone therapy		
Yes	119	43%
No	158	57%

*UICC*, Union for International Cancer Control

#### Measures

Measures of subjective norms, perceived behavioural control, attitudes, and intentions from the TPB were adapted following Ajzen’s [27] recommendations for TPB construct and based on scales used in previous studies e.g. [40–42].

##### Subjective norms (SN)

Subjective norms were measured using six items rated on a 5-point Likert-type scale from 1 (strongly disagree) to 5 (strongly agree) adapted from a previous study [43]. A sample item is “People who are important to me want me to have COVID-19 vaccine when it becomes available”. The scale showed a good internal reliability, α = 0.90. Items were averaged to create the composite, with higher scores reflecting norms favouring vaccination.

##### Perceived behavioural control (PBC)

Perceived behavioural control was measured with four items rated on a 5-point Likert-type scale from 1 (strongly disagree) to 5 (strongly agree) adapted from a previous study [44]. A sample item is “I am confident that I will be able to get the COVID-19 vaccine when it becomes available is easy to me”. The scale showed a good internal reliability, α = 0.83. Items were averaged to create the composite, with higher scores reflecting greater confidence in one’s ability to obtain the vaccine.

##### Attitudes towards COVID-19 vaccination (ATV)

Attitudes towards COVID-19 vaccination were measured by three 5-point evaluative semantic differential words: “I think that to get the COVID-19 vaccine when it becomes available is bad-good, harmful-beneficial, useless-useful”, adapted from a previous study [45]. The internal reliability of the scale was excellent α = 0.97. Items were averaged to create the composite, with higher scores reflecting more positive attitudes towards vaccination.

##### Intention to get COVID-19 vaccine (ItoV)

Intention to get COVID-19 vaccine was measured with three items rated on a 5-point Likert-type scale from 1 (strongly disagree) to 5 (strongly agree), adapted from a previous study [46]. A sample item is “I intend to have the anti-COVID-19 injection when it becomes available”. The internal reliability was excellent, α = 0.98. Items were averaged to form the composite, with higher scores indicating stronger intentions to be vaccinated.

##### Perceived risk of COVID-19 (PRC-19)

Perceived risk of COVID-19 was measured by assessing respondents’ agreement on a 5-point Likert-type scale ranging from 1 (strongly disagree) to 5 (strongly agree), with four statements, such as “There is a high likelihood of dying from COVID-19”. This scale was adapted from a previous study [47]. The internal consistency of the scale was good α = 0.84. Items were averaged to form the composite, with high scores indicating greater risk perception.

##### Trust in health authorities (THA)

Trust in health authorities was measured using four items adapted from a previous study [45]. Each item (e.g. “The COVID-19 vaccination program is safe because it is approved by the Health Ministry”) was rated on a 5-point Likert-type scale ranging from 1 (strongly disagree) to 5 (strongly agree). The scale showed a good internal consistency, α = 0.96. Items were averaged to create the composite, with higher scores indicating greater trust.

##### Previous vaccination history for the seasonal vaccine (PVH)

Respondents were asked whether they had received any seasonal influenza vaccination in the past 5 years (yes/no).

##### Vaccination uptake (VU)

Data was collected six months after the baseline survey by asking participants whether they had completed the vaccination against COVID-19 (yes/no).

### Procedure

Psycho-oncologists (D. V.; M. V.; M. C.) working in the Italian Voluntary Association “Ali Rosa” provided contact information for eligible patients. A cross-sectional web-based survey design was adopted using the free software Google Forms®. From January 29th to February 28th of 2021, online recruitment targeted all potential Italian participants. A study invitation letter that included the research purposes and the link to the online questionnaire was mailed to all patients (T0). The response rate was 48%.

The participants were informed that their participation in the research was voluntary and confidential, and they could withdraw from the study at any time; all completed an online consent form. Finally, participants were invited to participate in a follow-up survey on vaccination uptake 6 months later (T1). The response rate was 52.5%. Approval for this study was obtained from the Ethical Committee of Calabria Region (Catanzaro, Italy).

### Statistical analysis

All the variables were screened for univariate normality (skewness and kurtosis) and multivariate outliers by computing Mahalanobis distance [48]. There were no missing data since answers to the items were required to proceed in the online survey. Following the general recommendations for univariate normality, all skew and kurtosis values were within ± 2.0. The greatest skew was seen for attitude towards COVID-19 (− 1.25) and intention to vaccination (− 1.22), and all values for kurtosis were < 1.0. The results of the Mahalanobis distance with *p* < 0.001 for the chi-square (χ^2^) indicated no multivariate outliers.

Descriptive statistics were calculated for all study variables, and correlational analyses (Pearson’s) were run to examine the bivariate relationships among the variables. The reliability of the main variables was tested using Cronbach’s alpha (α) coefficient. According to Kline [49], reliability was considered “excellent” for values of Cronbach’s α 0.90, “good” for α between 0.90 and 0.80, and “acceptable” for α between 0.80 and 0.70.

The preliminary data analysis was run with SPSS 26; structural equation modelling (SEM) and mediational analysis were performed using Mplus 7.04 [50]. According to Hu and Bentler [51], multiple fit indices were used to evaluate the goodness of the SEM model (adopted cut-offs in brackets): the chi-square (χ^2^) test value with the associated *p* value (*p* > 0.05), comparative fit index (CFI ≥ 0.90), Tucker–Lewis index (TLI ≥ 0.90), root mean square error of approximation (RMSEA ≤ 0.06), and its 90% confidence interval, and standardised root mean square residual (SRMR < 0.08).

The SEM model was estimated with the weighted least square mean and variance-adjusted (MLM), maximum likelihood parameter estimates with standard errors, and a mean-adjusted chi-square test statistic robust to non-normality [50]. A mediation analysis was conducted to examine the potential mediating role of the TPB variables between the variables of perceived behavioural control, perceived risk of COVID-19, trust in health authorities, and the outcome of intention to receive a COVID-19 vaccination. Partial and full mediational models were compared using a chi-square (χ^2^) difference test, which provides a statistical indicator of whether the constraints are justified (Kline, 2016). According to Chen [52], a more parsimonious model requires that at least two out of three criteria be satisfied: Δχ^2^ significant at *p* < 0.05, ΔCFI ≤ 0.005, and ΔRMSEA ≤ 0.010. All analyses were controlled for age, gender, and previous seasonal influenza vaccination history.

## Results

### Descriptive and correlations

The results of the correlational analysis, controlling for age and seasonal vaccination history, indicated that most of the variables were positively associated (Table 2). Of all the variables, the means for attitude towards COVID-19 vaccination (ATV) and the intention to get a COVID-19 vaccine (ItoV) were the highest, suggesting that this group’s scepticism and hesitancy were low a priori. This result suggests that participants might be more likely to have a coronavirus vaccination. It is also noteworthy that participants reported considerable trust in Italian health authorities, subjective norms in favour of getting the COVID-19 vaccine, and perceived behavioural control for getting the COVID-19 vaccine. At follow-up, 96.5% of respondents reported having received the COVID-19 vaccine.

**Table 2:** Descriptive statistics and correlations between the main variables of the study

	*M*	*SD*	1	2	3	4	5	6	7	8
1. PRC-19	3.12	.99	-							
2. THA	3.66	1.22	.15*	-						
3. SN	3.84	1.14	.25***	.63***	-					
4. PBC	3.77	1.08	.09	.62***	.64***	-				
5. ATV	4.18	1.15	.16***	.80***	.74***	.73***	-			
6. ItoV	4.07	1.30	.19**	.75***	.81***	.75***	.89***	-		
7. Age	49	11.53	− .00	.08	.16**	.08	.08	.07	-	
8. PVH	1.30	1.73	.05	.26***	.26***	.22***	.24***	.29***	.27***	-

*PRC-19*, perceived risk of COVID-19; *THA*, trust in health authorities; *SN*, subjective norms; *PBC*, perceived behavioural control; *ATV*, attitude towards COVID-19 vaccination; *ItoV*, intention to vaccination; *PVH*, previous seasonal influenza vaccination history. *p < .05. **p < .01. ***p < .001

### Structural equation modelling

#### Measurement model

Based on Hu and Bentler [51], the fit of the measurement model, permitting the latent variables to freely correlate, showed an excellent fit to the data, robust χ^2^ [13, *N* = 277] = 15.80, *p* = 0.260, CFI = 0.997, TLI = 0.993, RMSEA = 0.028, 90% CI [0.000, 0.069], SRMR = 0.028.

#### Mediation analysis

**Fig. 2:**
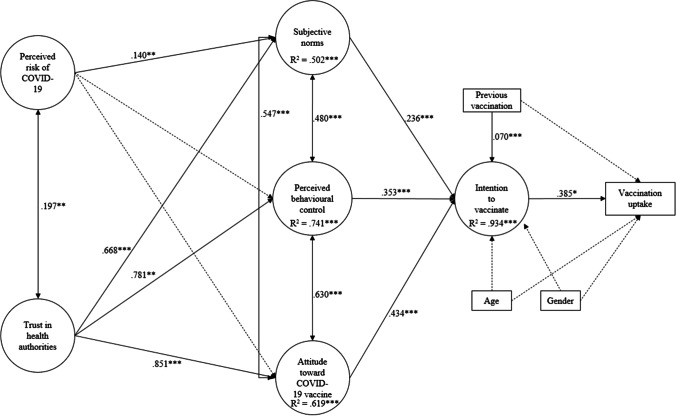
Standardised results of the partial mediation model including direct, indirect effects, and significance levels. Dashed lines represent nonsignificant relationships between variables. Note: *p < .05. **p < .01. ***p < .001. For clarity, item factor loadings, which were all significant at* p* < .001, are omitted

As mentioned above, first we tested the less parsimonious partial mediation model, performing both the structural and the measurement model simultaneously (Fig. 2). The results of the fit indices were good, robust χ^2^ [272, *N* = 277] = 332.21, *p* = 0.007, CFI = 0.980, TLI = 0.977, RMSEA = 0.028, 90% CI [0.016, 0.038], SRMR = 0.064. We also found that most of the direct paths and the mediated coefficients were significant (Table 3).

**Table 3:** Mediation analysis and direct and indirect effects with standardised results

Effects from THA to ItoV	Estimate	*SE*	*t*	*p*
Total	.790	.034	23.476	.000
Direct	-.030	.045	-.631	.528
Specific indirect effects				
ItoV—> SN—> THA	.147	.034	4.303	.000
ItoV—> ATV—> THA	.380	.085	4.491	.000
ItoV—> PBC—> THA	.291	.074	3.924	.000
Effects from CRP to ItoV	Estimate	*SE*	*t*	*p*
Total	.094	.044	2.138	.033
Direct	.042	.021	2.040	.041
Specific indirect effects				
ItoV—> SN—> PRC-19	.030	.014	2.158	.031
ItoV—> ATV—> PRC-19	.017	.019	.864	.388
ItoV—> PBC—> PRC-19	.005	.016	.323	.746

*ItoV*, intention to vaccination; *SN*, subjective norms; *ATV*, attitude towards COVID-19 vaccine; *PBC*, perceived behavioural control; *THA*, trust in health authorities; *PRC-19*, perceived risk of COVID-19

Subsequently, we tested the complete mediation model, by which the direct paths from the independent variables (perceived risk of COVID-19 and trust in health authorities) to intention to vaccinate were removed. The fit indices were again good, robust χ^2^ [274, *N* = 277] = 334.79, *p* = 0.007, CFI = 0.980, TLI = 0.977, RMSEA = 0.028, 90% CI [0.016, 0.038], SRMR = 0.064, and the difference in chi-square compared to the partial mediation model was nonsignificant, Δχ^2^ (2) = 3.45, *p* = 0.178. In addition, the variations of fit indices (ΔCFI = 0.000; ΔTLI = 0.000; ΔRMSEA = 0.000) were low. Considering that both partial mediation model and direct effects exhibited good fit, the more parsimonious solution was chosen, as suggested by Brown [53] (Fig. 2).

Regarding the relationships across perceived risk of COVID-19, trust in health authorities, and TPB, the current results show that trust in health authorities was a consistently significant and positive predictor of TPB variables, compared to the perceived risk of COVID-19 which was positively associated only with subjective norms. Therefore, for this sample of cancer patients, trust in health authorities is a key factor.

## Discussion

The Italian government has launched a national vaccination campaign against COVID-19, yet some people still refuse to be vaccinated, including cancer patients. The most common reasons for vaccine hesitancy documented in cancer patients are fear of the vaccine impact on cancer therapy or outcome [20], fear of side effects or vaccine safety [20–22], and lack of information or misconception [16, 23].

The aim of the present study was to explore the factors affecting the intention to get a COVID-19 vaccine among cancer patients, by integrating the theory of planned behaviour (TPB) and the Health Belief Model (HBM). Therefore, this study aimed to better understand the psychosocial constructs underpinning their intention to get a COVID-19 vaccine and their follow-through. The integrated model linked perceived risk of COVID-19, trust in health authorities, subjective norms, perceived behavioural control, attitudes towards COVID-19 vaccines, and intention to be vaccinated and, in turn, the association between the intention to be vaccinated and vaccination uptake. Data showed that the hypothesised model well fit the data.

Consistent with our hypotheses, the study results showed that cancer patients who had higher trust in health authorities tended to have vaccine-positive subjective norms, perceptions that vaccination was under their control, and more positive views on COVID-19 vaccines. In contrast, the perceived risk of COVID-19 was positively associated only with subjective norms. However, a direct and positive association was found between intention to vaccinate and the three psychological determinants of the TPB. Overall, hypothesis 1 was partially supported by the current data and the integrated and extended TPB model provided helpful insights for understanding which factors motivate people to engage in health-related behaviours [15, 26, 29]. However, in this study, trust in health authorities was the most important predictor of vaccine acceptance, and this result is consistent with a recent scoping review [39].

Additionally, the perceived risk of COVID-19 was not strongly associated with the intention to vaccinate in this sample of cancer patients. This result is partially in line with the expectations of the HBM health-related beliefs. Indeed, specific HBM factors that work best may vary across behaviours, indicating individual variability in perceived severity and risk [33]. However, this result might be due to various factors, including psychological dispositions, fear of side effects, and other related biased cognitions, as have been described elsewhere [34, 47]. For example, individuals might overestimate the vaccine’s side effects (e.g. particularly if anecdotal reports have been highlighted in the media) and underestimate the benefits of the vaccination in terms of protection from the infection (e.g. especially if one knows someone who was vaccinated but still became ill). Moreover, there was no direct association between trust in health authorities and intention to vaccinate in this sample. This is interesting and suggests that this trust might be an essential foundational element, but it is likely not enough to convince cancer patients that vaccinations are necessary. Instead, this trust can be best leveraged in the context of subjective norms, attitudes towards the vaccine, and perceived behavioural control.

Regarding the mediation effects, we found that only subjective norms partially mediated the relationship between the perceived risk of COVID-19 and intention to vaccinate (hypothesis 2 was partially supported). On the other hand, we found a full mediated effect of trust in health authorities and intention to vaccinate (for this association, hypothesis 2 was supported). Specifically, the current results indicated that attitude towards COVID-19 was the strongest mediator of intention to be vaccinated, and then perceived behavioural control and subjective norms were the weakest. Moreover, it indicates that the effort to encourage anti-COVID-19 vaccination would fall short if communication strategies do not consider the importance to underline the essential role of trust in health authorities to increase health-protective behaviour. This result was consistent with previous studies related to behavioural change [36, 45]. Therefore, the three determinants of the TPB differently mediated the relationship between antecedents and intention to vaccinate. The current study reveals that TPB variables can function effectively as mediators between perceived risk, trust, and intention to vaccinate but at different levels. Finally, a positive and significant association was found between intention to vaccinate and vaccination uptake (supporting hypothesis 3).

The present study has two main implications. First, subjective norms were quite related to intention to vaccinate, indicating that significant others need to provide more and more evidence regarding the safety and effectiveness of COVID-19 vaccines and emphasise the benefits of being vaccinated. Thus, ensuring that these significant others are well-informed and confident may do much to shift intentions among the target group, and empowering patients themselves to share their positive views (in person or via social media) regarding vaccinations may also be an effective strategy. Second, attitude towards the COVID-19 vaccine was strongly related to the intention to vaccinate, which means that interventions aimed at shifting attitudes in positive ways (e.g. through written materials or conversations with healthcare providers) may meaningfully impact vaccination intentions.

Nevertheless, this research has several limitations that warrant mention. First, the non-random nature of the sample increases the likelihood of bias, so a random sampling approach (or, if that is impossible, a better assessment of potential confounding variables) would enhance the internal validity of future studies. Second, although the present sample is varied in its characteristics and not small, an even larger sample would more closely approximate the population. Third, the current sample of cancer patients come from one country, Italy, and the results may not generalise to other nations, as cultural norms related to COVID-19, vaccination, and patient exposure to anti-vaccine media messages may be distinctive among different nations. Finally, a longitudinal design would be ideal to understand better the relationships of these variables over time and through the cancer progression.

## Conclusion

In conclusion, the present study tested a prospective model to understand better the intention to get the COVID-19 vaccine among cancer patients. Cancer patients reported considerable trust in Italian health authorities, subjective norms positive towards the vaccine, and perceived behavioural control over vaccine acquisition. Furthermore, although there was no direct association between trust in health authorities and vaccine intentions, TPB variables served as indirect pathways between the two. Therefore, vaccine policy should engage systematic and strategic programs to enhance trust in health authorities while at the same time sharing information regarding the safety and efficacy of the COVID-19 vaccine (both of which strengthen vaccine-positive subjective norms) and making clear how cancer patients can access vaccines. It may also be helpful to facilitate a more accurate understanding of the risks of COVID-19 among cancer patients.

## Data Availability

The dataset generated of the current study is available from the corresponding author on reasonable request.

## Code availability

The statistical syntax and the code generated of the current study are available from the corresponding author on reasonable request.
